# A Health Equity–Oriented Research Agenda Requires Comprehensive Community Engagement

**DOI:** 10.2196/37657

**Published:** 2022-09-30

**Authors:** Kevin Rice, Joshua Seidman, Oneil Mahoney

**Affiliations:** 1 Fountain House New York, NY United States; 2 Columbia University New York, NY United States

**Keywords:** mental health, community-based participatory action research, health equity, serious mental illness, health policy, research, community, engagement, disparity, participatory care, consumers

## Abstract

Health policy and research communities have taken new approaches to addressing health equity, going beyond traditional methods that often excluded the contributions of health care consumers and persons with lived experience. This reevaluation has the potential to drive critical improvements in how we conduct research and innovate policy toward reducing health and health care disparities in the United States. Such considerations have led Fountain House, the founder of the Clubhouse model for peer-based psychosocial rehabilitation for persons with histories of serious mental illness, to incorporate community-based participatory action research (CBPAR) protocols within their research and service programs. The combination of CBPAR research methods within novel participatory care settings like Clubhouse programs presents unique and informative opportunities for the advancement of innovative health equity approaches to consumer empowerment in health care. In this piece, the authors (two staff researchers and one member researcher) propose how CBPAR research methods conducted in Clubhouses can uniquely advance equity-focused research methods, and how the benefit and enhancements from equity-focused research are continuously applied, practiced, and accountable to the communities within which the research is conducted. Embedding CBPAR practices within participatory care settings like Clubhouses, creates novel opportunities for research work to not only become more equitable but also become a part of the rehabilitative process, empowering the main beneficiaries of the research with the means to sustain and achieve further improvements for themselves. Such experiences are particularly important within rehabilitation settings, where there is a process of reclaiming empowerment and self-efficacy over a disability or illness and the social circumstances surrounding those conditions. Different stakeholders can all play important roles in advancing health equity–oriented research agendas by leveraging CBPAR principles. Academics and others in the research community can more comprehensively embed CBPAR methods into the design of their research studies. A critical link exists among how researchers conduct their studies, how providers organize care delivery and support, and how health plans pay for and evaluate care. CBPAR-generated research needs to fully engage clinical teams to ensure that ongoing community-involved care settings have direct applications to real-world care delivery. It is equally important that providers fully engage with their communities as they adjust their approaches to supporting the populations they serve.

## Introduction

In recent years, health policy and research communities have adapted and rethought traditional approaches to health equity that often excluded the contributions of health care consumers and persons with lived experience. This reevaluation has the potential to drive critical improvements in how we conduct research and innovate policy toward reducing health and health care disparities in the United States, consistent with the recent 2021 call to action by the Robert Wood Johnson Foundation (RWJF) “for its health equity agenda, the Biden administration needs research that focuses on impacted communities” [1]. Moving this equity agenda forward requires providers and researchers to fully embrace participatory strategies with the communities they serve in both the conduct of participatory research and in the development of participatory care environments, where research benefits can persist within.

Such considerations have led Fountain House, the founder of the Clubhouse model for peer-based psychosocial rehabilitation for persons with histories of serious mental illness (SMI), to incorporate community-based participatory action research (CBPAR) protocols within their research and service programs. Central to the Clubhouse model is the joint operation of its services by professional staff working side by side with Clubhouse members (people with an SMI who join the Clubhouse have always been called members rather than patients or clients) in all aspects of Clubhouse program operations. Clubhouses intentionally structure therapeutic experiences and growth through the shared work of Clubhouse programs, emphasizing socialization and member empowerment to combat loneliness and stigma while also connecting members to traditional health and social support services.

Recognizing the synergy between CBPAR research methods with participatory care settings like Clubhouse programs presents unique and informative approaches to the advancement of health equity–focused research that involves consumer empowerment and continuous participation. In this piece, the authors (two staff researchers and one member researcher) propose how CBPAR research methods conducted in Clubhouses can uniquely advance equity-focused research and how the benefit and enhancements from this research are continuously applied, practiced, and accountable to the communities within which the research is conducted.

## Defining CBPAR

CBPAR starts with the principle that all aspects of research should involve true collaboration among professional researchers and community of interest stakeholders, or colloquially, it holds true to the mantra “nothing about me without me.” Various formal definitions and approaches of CBPAR and community-based participatory research (CBPR) have been advanced. We embrace the same definition as used in the Chicago Health Disparities Study adapted from the WK Kellogg Foundation’s Community Health Scholars: “CBPR is a collaborative approach that involves all partners in the research process. [It] begins with a research topic of importance to the community...[combining] knowledge and action for social change to improve communities and eliminate disparities” [2].

Going beyond aligning research with community priorities and experiences, CBPAR methods also prioritize the training of community participants in scientific design and procedures so that the community can collaborate in research decision-making from a shared knowledge position. This level of participation and training empowers the community to leverage skills for continued assessment and advancement of the community’s interests beyond the scope and limitations of a given research study [3]. This is specifically relevant for the “action” processes of CBPAR, where research developed toward change-oriented solutions can be implemented, sustained, and enhanced on an ongoing basis within the communities where the research was conducted.

CBPAR methods have particular importance for communities of interest that have historically been marginalized from participating in larger social systems that impact their daily lives. Some prominent CBPAR practice examples have occurred within indigenous communities managing diabetes prevention resources [4], migrant communities accessing social service resources [5], and mental health communities seeking greater advocacy for addressing social determinants of health related to poor outcomes [6]. Rather than being a burden, the empowering benefits of such collaborative approaches often enhance research quality, demonstrating more realistic and practical results due to the introspective data and action-oriented decision-making provided by community stakeholders in research procedures [7,8].

## Health Equity and CBPAR

Health equity has been defined in multiple ways. Borrowing from RWJF, “health equity means that everyone has a fair and just opportunity to be as healthy as possible.” The RWJF definition further elaborates that health equity “requires removing obstacles to health such as poverty, discrimination, and their consequences, including powerlessness and lack of access to good jobs with fair pay, quality education and housing, safe environments, and health care.” Powerlessness and access barriers, in particular, have been parallel obstacles to equity in both health and health research [9].

In alignment with health equity goals, CBPAR methods offer unique research strategies to help address larger systemic issues related to health care accessibility, health literacy, and poor patient experiences [10]. However, certain change-oriented CBPAR outcomes can be complicated in many traditional public health settings, where imbalanced provider-patient power dynamics persist in terms of care decision-making, priority, and quality [11]. This power imbalance is particularly salient for persons with histories of SMI who can be forced to receive compulsory care within settings from which they are often disenfranchised.

Given such circumstances, while CBPAR methods can be readily used in traditional health settings, the persistent empowerment of patient communities to participate in the continuous change-oriented enhancements, delivery, and assessments of their own ongoing care is often limited [12]. To overcome these limitations, CBPAR and health equity agendas should pursue greater applications within a broader community-oriented approach to health care delivery that incorporates participatory practices in their core service model.

## The Clubhouse Model: Maximizing Participatory Potential for Health Equity

Although minimal in their overall presence in health care, there are some rehabilitation settings that operate unique care models focused on uplifting consumers into roles of treatment decision-making and peer-support delivery. A historical leader in such approaches is the Clubhouse model, a community-based psychosocial rehabilitation program for persons with histories of SMI. Founded in 1948 by persons with an SMI, the New York City–based Fountain House launched the Clubhouse movement with the purpose of creating communities of lived experience, where persons with histories of SMI could support and care for one another in their recovery journey.

Clubhouse programs offer strength-based peer interventions to help persons with SMI socially reintegrate and achieve agency in their health, quality of life, and care. This is achieved through the creation of an intentional peer community, where members are invited to (co)operate and administer Clubhouse operations, working side by side with Clubhouse professional staff to either receive or provide a range of social support services [13]. Member participation in Clubhouse services occurs through a structured work-ordered day, where members participate in the administration and delivery of peer-based support programs that often include education, care management, research, wellness, employment, and housing [14]. These facilitated experiences of shared contribution and administration drive what the Clubhouse calls the need to be needed, rehabilitating member agency, self-confidence, skills, and social acceptance, which have often been disrupted by shared histories of disenfranchisement, stigma, and diminished quality of life opportunities [15]. At the core of the Clubhouse model, every program decision, activity, and service offering involves member contributions, decision-making, and administration to the benefit of not only the consumers of Clubhouse services but also the member stakeholders who jointly run the Clubhouse alongside professional staff.

Recognizing the participatory congruence between Clubhouse model and CBPAR research methods, Fountain House has sustained a legacy of incorporating CBPAR practices in its research initiatives. Programmatically, this has taken the form of Fountain House maintaining a longstanding Research Unit as one of its program service areas, where members learn and direct the community’s research interests and priorities. Members and staff have also created a Research Committee to manage high-level administrative decisions in developing research collaborations across the national Clubhouse network and public health policy agenda. These continuous peer-led research forums empower members to develop research skills, translate their research priorities, and self-administer the change-oriented outcomes of their research toward positive program enhancements in their own care settings. The opportunities, insights, and skills developed through CBPAR within peer-driven programs like Clubhouse allows not just for health equity research advancements to be discovered but for them to also be accountably enacted and implemented by the very people whom they are intended to benefit within the settings they help operate.

One example of this unique CBPAR health equity dynamic within Clubhouse care settings occurred in a collaboration between Fountain House and Yale University, where Clubhouse members were trained in qualitative research to conduct an analysis of member care experiences, trajectories, and differing needs within the community. The results of this study, conducted from start to finish by members, informed programming decisions around new member orientation and needs assessment procedures that seek to engage members during “critical periods” of early membership, identifying a spectrum of member experiences interacting with the Clubhouse as either a supportive stepping stone or a long-term community destination. The members who administered the study were able to inform new program practices and further apply their research training toward training other members and even work as paid research consultants and coders in future research collaborations. This has been the case with a current CBPAR project with Harvard Medical School to co-design a virtual healthy lifestyle intervention that seeks to involve members not only in the development and implementation of the research study but also in administering components of the intervention themselves, after the study’s completion. What we have seen from this approach with Harvard, in addition to other CBPAR projects, is that members of different racial, social, and health backgrounds actively engage and adjust interventions and research protocols that address their collective needs, thus driving more equitable care support approaches that they can supervise and perform continuously.

By embedding CBPAR practices within participatory care settings like Clubhouses, the research work not only becomes more equitable but also restorative, empowering the individuals who are meant to benefit from the research with the means to achieve that improvement for themselves. This is especially important within rehabilitation settings, whereby in virtue of being in need of rehabilitation, there is a drive to reclaim empowerment and self-efficacy not only over a disability or illness but also the social circumstances surrounding those conditions. This has been the experience of one of the authors, who took an academic leave for mental health reasons, engaged with the CBPAR program at Fountain House, and is now undertaking academic pursuits toward developing strategies for consumer-informed solutions within the mental health industry.

## Moving Forward: Advancing a Health Equity Research Agenda

Different stakeholders can all play important roles in advancing health equity–oriented research agendas by leveraging CBPAR principles. Academics and others in the research community can more comprehensively embed CBPAR methods into the design of their research studies. Although the National Institutes of Health has a CBPAR program in its National Institute on Minority Health and Health Disparities, it has not built CBPAR requirements into its standard grantmaking process. National Institutes of Health and other federal agencies can create more robust expectations for applying researchers and support the promotion of community health settings, where CBPAR practices can be fully enacted for the continued benefit and empowerment of patient communities and their role in care design and delivery.

How researchers conduct their studies will benefit from greater coordination with and application within participatory care settings. To further enhance health equity–focused research approaches, CBPAR-generated research needs to fully engage clinical teams and consumer communities to ensure that ongoing community-involved care settings have direct applications to real-world care delivery. This is particularly important for exploring and promoting innovations in how health plans pay for certain types of care. Indeed, providers are unlikely to shift their models unless health plans and other payers embed community-based participation and human-centered design into their payment models. The final piece of this effort relates to the role that state and federal policy making has on this intersection between health equity and community-oriented research models. Policy makers—those involved in both legislative and regulatory aspects—need to fully embrace CBPAR as one of several vehicles for advancing a national agenda to promote health equity, which includes the investment and promotion of participatory and peer-driven treatment settings where CBPAR methods can be implemented. Ultimately, how we measure and improve health equity will be dramatically influenced by the research questions we ask and the way we conduct that research. The communities we want to support must be integrally involved.

## Abbreviations

CBPAR: community-based participatory action research
CBPR: community-based participatory research
RWJF: Robert Wood Johnson Foundation
SMI: serious mental illness
